# Advancements and challenges in pharmacokinetic and pharmacodynamic research on the traditional Chinese medicine saponins: a comprehensive review

**DOI:** 10.3389/fphar.2024.1393409

**Published:** 2024-05-07

**Authors:** Yuhan Ma, Yongxia Zhao, Mingxia Luo, Qin Jiang, Sha Liu, Qi Jia, Zhixun Bai, Faming Wu, Jian Xie

**Affiliations:** ^1^ School of Pharmacy, Zunyi Medical University, Zunyi, China; ^2^ Organ Transplant Center, Affiliated Hospital of Zunyi Medical University, Zunyi, China; ^3^ School of Preclinical Medicine, Zunyi Medical University, Zunyi, China

**Keywords:** saponins, traditional Chinese medicine, pharmacokinetics, bioavailability, influencing factors

## Abstract

Recent research on traditional Chinese medicine (TCM) saponin pharmacokinetics has revealed transformative breakthroughs and challenges. The multicomponent nature of TCM makes it difficult to select representative indicators for pharmacokinetic studies. The clinical application of saponins is limited by their low bioavailability and short half-life, resulting in fluctuating plasma concentrations. Future directions should focus on novel saponin compounds utilizing colon-specific delivery and osmotic pump systems to enhance oral bioavailability. Optimizing drug combinations, such as ginsenosides with aspirin, shows therapeutic potential. Rigorous clinical validation is essential for practical applications. This review emphasizes a transformative era in saponin research, highlighting the need for clinical validation. TCM saponin pharmacokinetics, guided by traditional principles, are in development, utilizing multidisciplinary approaches for a comprehensive understanding. This research provides a theoretical basis for new clinical drugs and supports rational clinical medication.

## 1 Introduction

Saponins are distinguished by their intricate glycoside structures, comprising glycone units bonded to aglycones, either triterpenes or spirostanol. These compounds are prevalent across a wide range of organisms, spanning terrestrial (Papadopoulou et al., 1999), marine (Xiao et al., 2019), certain fungal (Jin et al., 2017), insect (Laurent et al., 2003), and even human intestinal microbiota (Guo et al., 2019). In the field of traditional Chinese medicine (TCM), notable sources include *Panax ginseng C.A. Meyer* (Xu et al., 2022), Gynostemma pentaphyllum (*Thunb.*) *Makino* (Shi et al., 2012), Polygala tenuifolia *Willd.* (Qiu et al., 2022), *Platycodon grandiflorus (Jacq.) A. DC* (Kim et al., 2018), *Glycyrrhiza uralensis Fisch* (Jain et al., 2022), *Anemarrhena asphodeloides* (Han et al., 2018), and *Bupleurum chinense* (Lim et al., 2021), underscoring their significance as principal bioactive ingredients.

Classified by their aglycone skeletons and sugar chain linkages, saponins include triterpenoid, spirostanol, steroidal, and fatty acid variants (Vincken et al., 2007). Notably, triterpenoid saponins are further categorized into tetracyclic, pentacyclic, and hexacyclic subtypes based on their aglycone rings (Connolly and Hill, 2008), each demonstrating unique chemical structures and biological activities. These activities encompass a spectrum from antibacterial (Jin et al., 2017) and anti-inflammatory/antioxidant (Chang et al., 2021) to blood pressure modulation, antidiabetic (Jovanovski et al., 2020), antipyretic, sedative, and anticancer properties (Sharma et al., 2021). The solubility of saponins, which is pivotal in dictating their pharmacokinetic profiles, plays a crucial role in their absorption, distribution, metabolism, and excretion (ADME) processes within the body (Zheng et al., 2023).

With its focus on specific disease markers, Western medicine occasionally leads to a high rate of adverse reactions and a lack of customized treatment options (Che et al., 2022). TCM, in contrast, offers a holistic approach by providing a diverse array of chemical constituents, including saponins, alkaloids, polysaccharides, enzymes, and therapeutically beneficial compounds. In addition, it supplies nutritionally advantageous substances such as proteins, amino acids, lipids, trace elements, and vitamins. The advantage of TCM lies in its multifaceted, multitarget approach, which helps counteract the adverse effects that genetic variability can have on the efficacy of Western medications (Jia et al., 2020). This multitarget strategy is especially relevant amidst the challenges of developing effective Western treatments for various diseases, such as gastric cancer. TCM has shown considerable potential in addressing these challenges by seamlessly integrating traditional formulas with specific herbal components (Xu et al., 2022). A particularly promising area of TCM application is in treating myocardial ischemia‒reperfusion injuries, where saponins from Chinese herbs have shown protective effects in both *in vivo* and *in vitro* studies. These effects are largely ascribed to their capacity to regulate energy metabolism, ensure calcium homeostasis, and mitigate oxidative stress and inflammation (Zhu et al., 2017; Wang et al., 2019). Additionally, in the treatment of cardiovascular disease (CVD), where conventional therapies often fail to reverse abnormal vascular remodeling (VR) or improve vascular function, ginsenosides, a specific type of saponin, have emerged as promising candidates to alleviate vascular dysfunction and correct VR, particularly in hypertension scenarios (Zhu et al., 2021). This context underscores the need for an in-depth investigation into the pharmacokinetic properties of saponins, which is crucial for elucidating their therapeutic potential and enhancing their clinical efficacy.

Pharmacokinetics, rooted in kinetic principles and mathematical modeling, is paramount for elucidating the trajectory of drugs within the human body. This discipline quantitatively describes the absorption, distribution, metabolism, and excretion (ADME) processes of drugs via various administration routes, such as intravenous injection, intraperitoneal injection, and oral administration (Hochhaus et al., 2000). TCMs are known for their complex chemical compositions. Investigating the pharmacokinetic-pharmacodynamic (PK-PD) relationships of TCMs enables the identification of key compounds responsible for therapeutic effects through bioavailability at the site of action and significant exposure levels postadministration (Li, 2017). Cardiotonic pills, a cardiovascular TCM, exemplify this approach. Research into suitable pharmacokinetic (PK) markers for systemic exposure to cardiotonic pills and the *in vivo* PK properties of presumed active phenolic acids derived from the component herb *Radix Salviae* miltiorrhizae—including tanshinol (TSL), protocatechuic aldehyde (PCA), salvianolic acids A, B, and D, rosmarinic acid, and lithospermic acid, demonstrating that plasma and urinary TSL are promising PK markers for cardiotonic pills at tested dosage levels (Lu et al., 2008). The Huanglian Jiedu Granule (HEJG), recommended in China for treating H1N1 influenza virus infection and COVID-19, is often coadministered with various therapeutic drugs in clinical settings. Studies indicate that coadministration of HEJG with lopinavir (a CYP3A substrate drug) in rats significantly increased the plasma exposure of lopinavir by 2.43-fold and extended its half-life by 1.91-fold through the inactivation of CYP3A, thereby modulating the pharmacokinetics of CYP substrate drugs (Zhang et al., 2022). A human pharmacokinetic study on XueBiJing, assessing the PKC of a XueBiJing/antibiotic combination, revealed that the inhibition of aldehyde dehydrogenase by seven antibiotics could reduce XueBiJing’s exposure to protocatechuic acid. XueBiJing/antibiotic coadministration demonstrated high PK compatibility at clinically relevant doses, offering a methodology for studying other drug combinations (Li et al., 2019). A comprehensive understanding of the benefits and limitations of translational pharmacokinetics is therefore essential. Such knowledge can not only improve the efficacy of clinical trials but also increase the likelihood of success in developing effective stroke therapies. In light of these examples, the study of pharmacokinetics, particularly in the context of TCM and its active components such as saponins, has become even more pertinent. This approach promises to unravel the complex ADME characteristics of these compounds, paving the way for more effective and safer therapeutic applications.

In the field of pharmacokinetics, understanding the ADME characteristics and mechanisms of active ingredients in TCM is as essential as understanding chemical drugs. The rich theoretical foundation and diverse resources of TCM place it at the forefront of innovative drug research in China. Early-stage pharmacokinetic investigations of potential TCM constituents are crucial for improving the success rate and efficiency of new drug development in this field. In recent years, there has been a surge in experimental and review studies focusing on saponins, including the review by Wang et al. on the pharmacological mechanisms of saponin components in TCM for the treatment of Parkinson’s disease (Wang et al., 2022). Chen et al. provided a comprehensive analysis of the antitumor activity of saponins, laying a theoretical foundation for their utilization in TCM (Chen et al., 2023). Daniel et al. explored the potential of saponins as antiviral agents (Mieres-Castro and Mora-Poblete, 2023), while Timilsena et al. investigated the chemical properties, functionalities, and regulatory aspects of saponins in food applications (Timilsena et al., 2023).

Despite these advancements, the literature still lacks a comprehensive summary of saponin pharmacokinetics. This review endeavors to bridge this gap by offering an exhaustive overview of the latest developments in saponin pharmacokinetics. This study aimed to elucidate the mechanisms of action, metabolic pathways, metabolites, and pharmacokinetic parameters of saponins, as well as the regulatory factors affecting their pharmacological activity. Furthermore, this review scrutinizes the existing challenges and limitations within this domain, providing insights into prospective research avenues and the future landscape of saponin pharmacokinetics.

## 2 Sources and pharmacological effects of saponins

### 2.1 Sources of saponins

#### 2.1.1 Plant-derived saponins

Saponins, a diverse group of phytochemicals, are broadly distributed across the plant kingdom, notably within specific families such as Araliaceae, Fabaceae, Campanulaceae, Gentianaceae, Apiaceae, and Ranunculaceae (Baky et al., 2022). Renowned for their wide-ranging pharmacological properties, these compounds are found in several key medicinal plants. For instance, ginsenosides extracted from *P. ginseng* are known for their adaptogenic effects. Saikosaponins from Bupleurum species, astragalosides from Astragalus, and hederagenin glycosides from *P. grandiflorus* represent other notable examples, each contributing unique therapeutic potential to the pharmacopeia of TCM (Du et al., 2018; Cheng et al., 2019; Wang et al., 2021). Moreover, steroidal saponins, primarily consisting of spirostanes and related steroidal structures, are prevalent in plants from the Liliaceae, Sapindaceae, and Dioscoreaceae families, highlighting the structural diversity and widespread occurrence of saponins in nature (Table 1) (Dong et al., 2019).

**TABLE 1:** Plant-derived saponins.

Description	Formula	Saponin name	R_1_	R_2_	R_3_	R_4_	Reference
Ginsenoside	Ginseng Diol 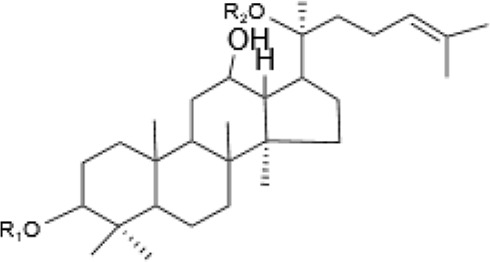	GinsenosideRb_1_	glc (2–1)glc	glc (6–1)glc	—	—	Cheng et al. (2019), Jeon et al. (2020), Qi et al. (2010), Tong et al. (2022), Zhang et al. (2022)
		GinsenosideRb_2_	glc (2–1)glc	glc (6–1)ara(p)	—	—	
		GinsenosideRb	glc (2–1)glc	glc (6–1)glc	—	—	
		GinsenosideRh_2_	glc (2–1)glc	glc	—	—	
	Ginseng triol 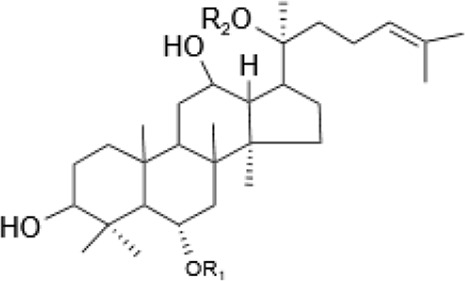	GinsenosideRe	glc (2–1)Rha	glc	—	—	
		GinsenosideRr	glc (2–1)glc	H	—	—	
		GinsenosideRg_1_	glc	glc	—	—	
		GinsenosideRg_2_	glc (2–1)Rha	H	—	—	
		GinsenosideRh_1_	glc	H	—	—	
	Oleanolic acid ginsenosides 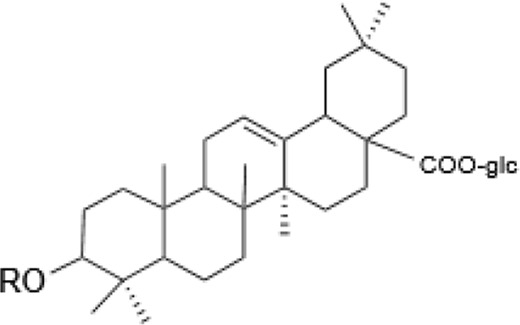	GinsenosideR_0_	glc (2–1)glc		—	—	
Saikosaponins	Oleanane triterpenoid saponinsⅠ 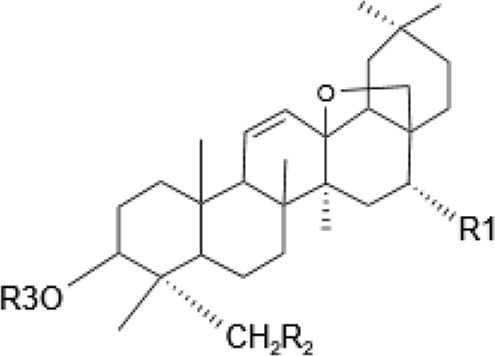	Saikosaponin a	β-OH	OH	β-D-glc (1–3)β-D-fuc-	—	Liu et al. (2021), Wang et al. (2021b), Yamamoto et al. (1975)
		Saikosaponin d	α-OH	OH	β-D-glc (1–3)β-D-fuc-	—	
		Saikosaponin e	β-OH	H	β-D-glc (1–3)β-D-fuc-	—	
		Saikosaponin c	β-OH	H	β-D-glc (1–6)/β-D-Rha (1–4)β-D-glc	—	
	Oleanane triterpenoid saponins Ⅱ 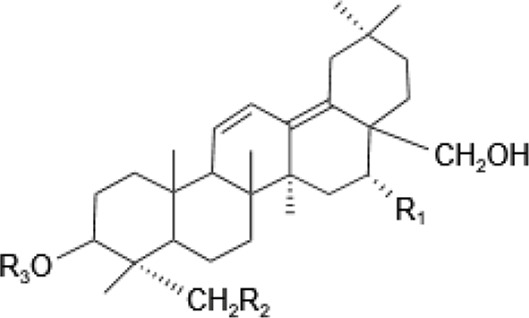	Saikosaponin b_1_	β-OH	OH	β-D-glc (1–3)β-D-fuc-	—	
		Saikosaponin b_2_	α-OH	OH	β-D-glc (1–3)β-D-fuc-	—	
	Oleanane triterpenoid saponins Ⅲ 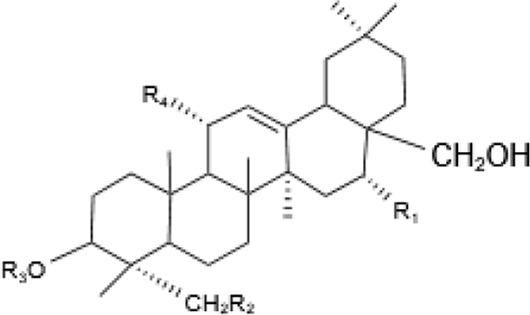	Saikosaponin b_3_	β-OH	OH	β-D-glc (1–3)β-D-fuc-	OCH_3_	
		Saikosaponin b_4_	α-OH	OH	β-D-glc (1–3)β-D-fuc-	OCH_3_	
	Oleanane triterpenoid saponins Ⅳ 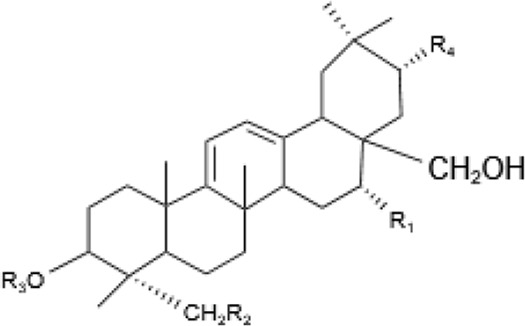	Saikosaponin g (Ⅳ)	β-OH	OH	β-D-glc (1–3)β-D-fuc-	H	
Astragaloside	triterpenoid saponin 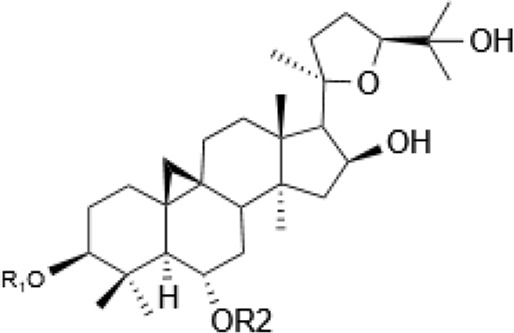	Acetylastragaloside Ⅰ	Xyl (2′,3′,4′-3OAc)	glc	—	—	Lee et al. (2013), Wang et al. (2013), Xing et al. (2018)
		AstragalosideⅠ	Xyl (2′,3′-2OAc)	glc	—	—	
		Isoastragaloside Ⅰ	Xyl (2′,4′-2OAc)	glc	—	—	
		Astragaloside	Xyl (2′-OAc)	glc	—	—	
		Astragaloside Ⅳ	xyl	glc	—	—	
		Astragaloside Ⅲ	Xyl (2–1)glc	H	—	—	
Diosgenin	Steroidal saponin 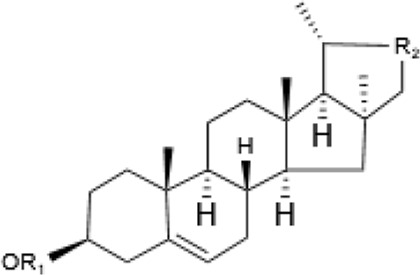	Diosgenin	H	C_6_H_12_O	—	—	Wang et al. (2022)
		Dioscin	glc (2–4)glc	C_6_H_12_O	—	—	

#### 2.1.2 Animal-derived saponins

**FIGURE 1:**
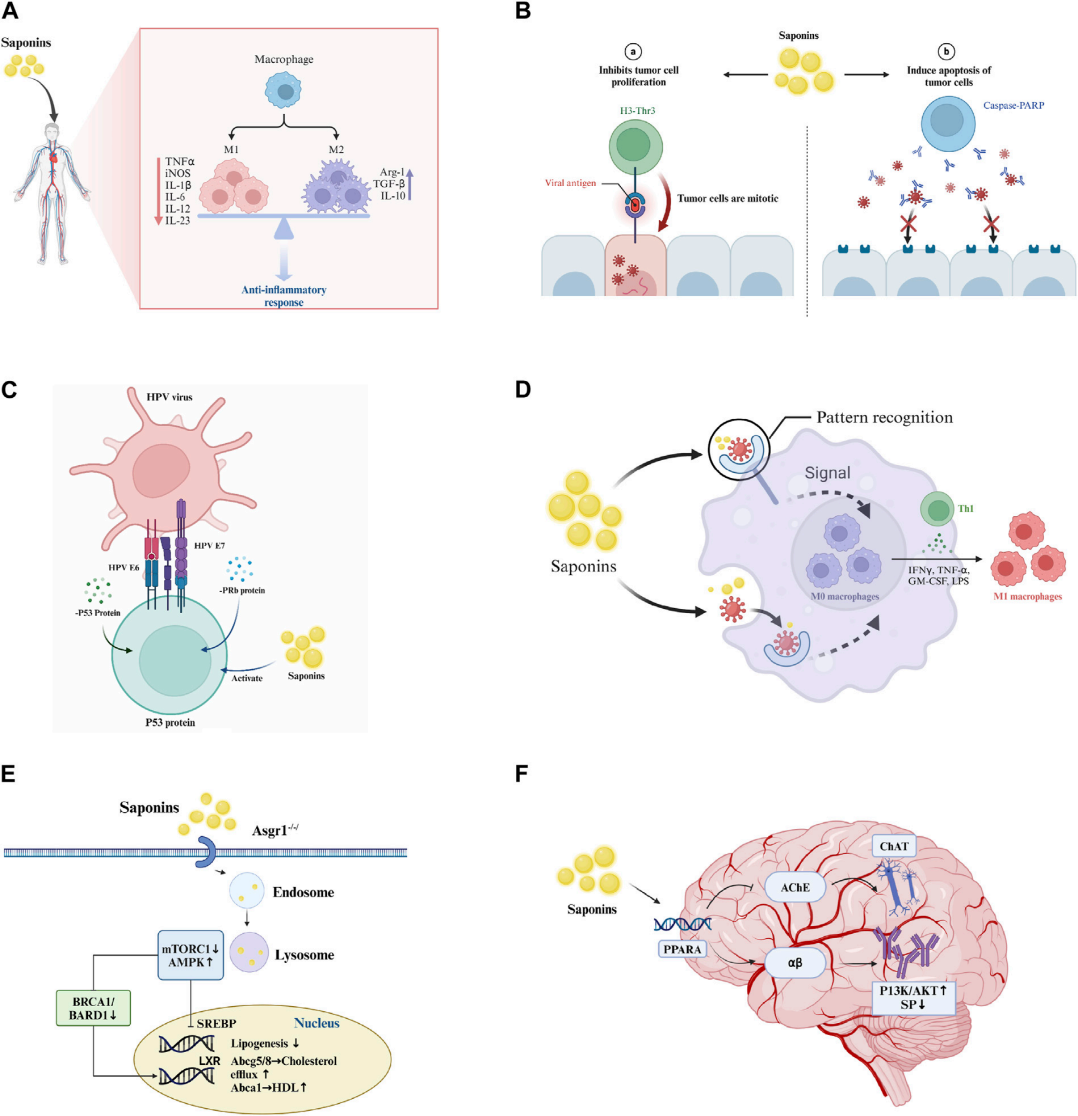
Pharmacological effects of saponins. **(A)** Anti-inflammatory effect: Saponins modulate immune cells to reduce inflammation by inhibiting the release of inflammatory mediators such as TNF-α and IL-1. **(B)** Antitumor effects: Saponins inhibit tumor cell proliferation and induce apoptosis through the activation of caspase-PARP pathways. **(C)** Antiviral effects: Saponins enhance immune response mediated by T-cells and B-cells, interfere with viral replication and propagation through the modulation of interferon p53 protein and PRb protein, and activate the immune system to augment antiviral efficacy. **(D)** Immunostimulation: Saponins boost the phagocytic activity of macrophages and promote the upregulation of cytokines and transcription factors associated with Th1 cells, including IFN-γ, TNF-α, and LPS. **(E)** Cholesterol reduction: Saponins regulate intracellular cholesterol levels through signaling pathways such as mTORC1 and AMPK. **(F)** Anti-Alzheimer’s: Saponins target the PPARA receptor, increasing acetylcholine concentrations in synaptic clefts to enhance neuronal signaling, influence the metabolism and aggregation of αβ-amyloid proteins, and reduce plaque formation, thereby slowing the progression of Alzheimer’s disease.

Within the animal kingdom, saponins are predominantly found in echinoderms, especially in Holothuroidea, a class of marine animals, including sea cucumbers (Kim and Himaya, 2012) (Table 2). These substances, which are secreted by the body wall and Cuvierian organs, are the primary secondary metabolites and serve as a chemical defense mechanism in sea cucumbers (Mondol et al., 2017). Additionally, saponins have also been identified in other marine organisms, such as sponges, soft corals, and certain species of small fish, underscoring the broad biological distribution and functional diversity of these compounds (Table 2) (Xiao et al., 2019) (Figure 1).

**TABLE 2:** Animal-derived saponins.

Description	Formula	Saponin name	R_1_	R_2_	Reference
Asterosaponin	Steroidal saponin 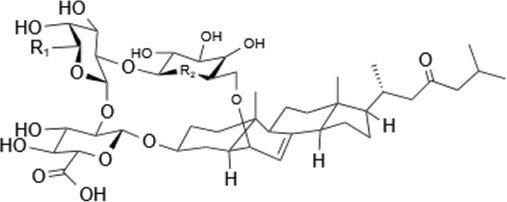	Luzonicoside A	ara	gal	Naruse et al. (2010), Xiao et al. (2019), Zhu et al. (2022)
		Luzonicoside D	ara	glc	
		Sepositoside A	aal	glc	
Sea cucumber saponin	triterpenoid saponin 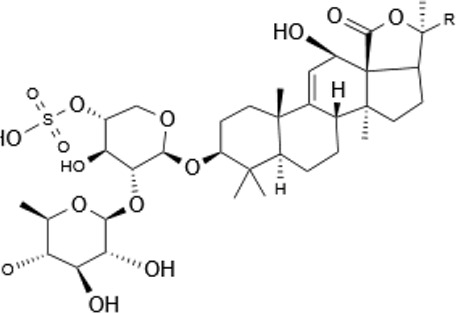	Echinoside B	C_5_H_12_	—	(Bahrami and Franco, 2016)
		Holothurin B	C_5_H_6_O	—	

### 2.2 Pharmacological effects and mechanisms of action of saponins

#### 2.2.1 Anti-inflammatory effects

Saponins have been recognized for their potent anti-inflammatory properties. Notable examples include ginsenosides from *P. ginseng*, saikosaponins from Bupleurum, and astragalosides from Astragalus, all of which have been shown to mitigate inflammation and associated conditions. Its mechanism of action primarily involves the inhibition of the nucleotide-binding oligomerization domain (NOD)-like receptor protein 3 (NLRP3) inflammasome, a key player in inflammatory responses (Yi, 2021; Li et al., 2023). Additionally, specific saponins, such as R1 from Panax notoginseng, have been shown to combat myocardial inflammation and apoptosis triggered by endotoxins through the upregulation of estrogen receptor alpha and the suppression of the NF-κB signaling pathway (Zhong et al., 2015). Akebia saponin D is another saponin known for its anti-inflammatory and cytoprotective effects (Gong et al., 2019). Moreover, saponin 29, derived from δ-oleanolic acid, significantly reduces the secretion of the proinflammatory cytokines TNF-α and IL-6 in macrophages activated by LPS (Liu et al., 2021).

#### 2.2.2 Antitumor effects

Saponins have also shown significant potential in combating cancer. Frondoside A, a sulfated saponin from sea cucumbers, inhibits the PAK1-dependent growth of lung and pancreatic cancer cells (Nguyen et al., 2017). Other saponins found in sea cucumbers, such as holotoxin A1 and holothurin A, display anticancer activity, making them valuable for functional food and nutraceutical applications (Song et al., 2017; Ru et al., 2023). A new sulfated saponin from sea cucumber exhibits antiangiogenic and antitumor activities both *in vitro* and *in vivo* (Hossain et al., 2020). Furthermore, a novel ginsenoside derivative, Rh_2(C-Rh_2), synthesized by Zhang et al., exhibits promising antitumor activity against H22 hepatoma-bearing mice, with lower toxicity than its parent compound (Zhang et al., 2019; Dai et al., 2020).

#### 2.2.3 Antiviral effects

Saponins have emerged as promising antiviral agents. Saikosaponin A (SSa) curtails the replication and infection of diverse type A influenza virus strains, including the highly pathogenic H1N3 strain, through the modulation of NF-κB and 5-lipoxygenase-dependent pathways (Chen et al., 2015). Similarly, Saikosaponin b2 significantly obstructs the early entry and infection stages of hepatitis C virus (HCV) in primary human hepatocytes (Lin et al., 2015). Moreover, the ginsenoside Rb1 has shown potent antiviral efficacy against enterovirus 71 (EV71), both *in vitro* and *in vivo*, serving as an effective immunostimulant for combating viral infections (Kang et al., 2021).

#### 2.2.4 Other biological activities

Saponins exhibit a diverse range of biological activities, including immunostimulation, cholesterol reduction, antibacterial, antifungal, and anti-Alzheimer’s disease effects (Rao and Gurfinkel, 2000). Arabski et al. observed a dose-dependent increase in early apoptotic cells and, notably, a rise in CFU/mL for multidrug-resistant *E. coli* strains in the presence of saponins derived from Quillaja (Arabski et al., 2012). Hederagenin from Lonicera fulvotomentosa Hsu et S.C. Cheng has been shown to significantly decrease the serum glutamic pyruvic transaminase and triglyceride levels in CCl4-intoxicated mice (Shi and Liu, 1996). Saponins such as Shubashin D (28) exhibit neuroprotective effects against Aβ25-35-induced cytotoxicity in PC12 cells, suggesting potential therapeutic applications against Alzheimer’s disease (Zhou et al., 2009). Ginsenosides have been noted for their role in reducing nerve damage leading to neurological disorders, including Alzheimer’s disease, Parkinson’s disease, depression, cognitive impairment, and cerebral ischemia, in addition to their therapeutic impacts on cardiovascular and cerebrovascular diseases and cancer (Chen et al., 2022). *Gynostemma pentaphyllum* saponins show anticancer, cardioprotective, hepatoprotective, neuroprotective, antidiabetic, antiobesity, and anti-inflammatory effects (Nguyen et al., 2021). Astragaloside has been found to inhibit proliferation and induce apoptosis in breast cancer cells, exhibiting varied effects across different cell types (Xu et al., 2023).

**FIGURE 2:**
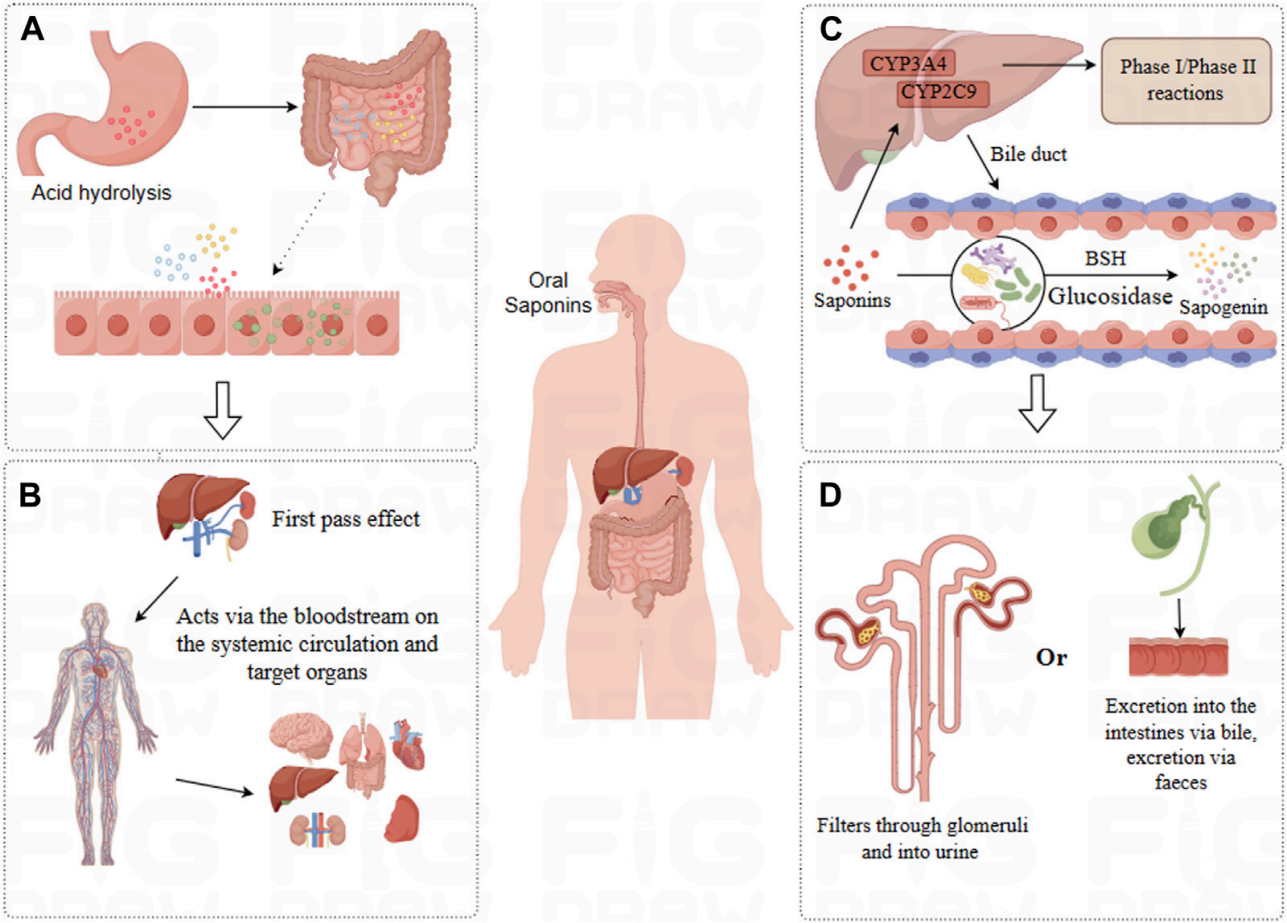
Pharmacokinetics of Saponins. **(A)** Absorption: Saponins undergo acid hydrolysis in the stomach, with a fraction directly absorbed in the small intestine, while others are transformed by the intestinal microbiota for absorption. **(B)** Distribution: Saponins are distributed via the bloodstream to various tissues, predominantly accumulating in organs such as the liver, kidneys, intestines, and spleen. **(C)** Metabolism: Saponins undergo Phase I and Phase II metabolic reactions in the liver, some compounds enter the intestines where they are metabolized by intestinal microbiota into more absorbable forms. **(D)** Excretion: Saponins and their metabolic products are primarily excreted through urine and bile.

According to comprehensive literature reports, most plant saponins have extensive pharmacological activity, and research on the pharmacological mechanisms of various saponins is relatively mature and has achieved the expected results. Therefore, TCM saponins have important medicinal value. However, from the overall research results, the difficulty in absorbing saponins in the body requires researchers to delve deeper. With the development of research technology, pharmacokinetic and other means can be used to explore the dynamic changes in drugs in the body, providing a basis for rational clinical medication. Despite in-depth research on the mechanism of action of saponins in the prevention and treatment of diseases, adverse reactions to saponins cannot be ignored, and their safety needs to be further evaluated. This will be more conducive to ensuring the safe, effective and rational application of saponins and will provide an important theoretical and scientific basis for the comprehensive development of their resources (Table 3) (Figure 2).

**TABLE 3:** Comprehensive pharmacokinetic profile and influential factors of saponins.

Saponin compound	ADME profile	Influential factors	Potential drug actions	References
Panax ginseng saponin	A: Only a minor portion of orally administered Panax ginseng saponin is absorbed directly in its original form. The majority undergoes hydrolysis in the gastrointestinal tract, resulting in the absorption of rare saponins and sapogenins	The chemical structure influences direct absorption into the bloodstream. Individual gastrointestinal tract capacities for saponin biotransformation are low, leading to reduced bioavailability	Panax ginseng saponin in combination with Apatinib significantly inhibits the proliferation, migration, and wound-healing ability of hypopharyngeal cancer cells. Coadministration with Warfarin significantly reduces its anticoagulant effect	Hasegawa (2004), Zhao et al. (2012), Qin et al., 2020; Li et al. (2022)
	D: Distributed throughout the bloodstream and various organs			
	M: Metabolized by gut microbiota through deglycosylation, followed by esterification with fatty acids			
	E: Excreted through the hepatobiliary or renal systems			
Panax notoginseng saponin	A: Cannot be absorbed in its original form, requires biotransformation	Poor membrane permeability and active biliary excretion limit systemic exposure	PNS and ASA (Aspirin) combination enhances ASA’s antiplatelet action via the AA/COX-1/TXB pathway and mitigates ASA-related gastric injury through the AA/PG pathway in the gastric mucosa	Liu et al. (2009), Xu et al. (2022b), Guo et al. (2020), Liu et al. (2012), Wang et al. (2021a)
	D: Distributed throughout the bloodstream and various organs			
	M: Undergoes deglycosylation in the intestines			
	E: Excreted via biliary pathways			
Astragalus saponin	A: Absorbed into the bloodstream post gastrointestinal hydrolysis	Low oral bioavailability is associated with various factors including physicochemical (i.e., solubility and dissolution) and physiological factors (i.e., intestinal absorption, efflux, and first-pass metabolism)	AST and Vinblastine (VBL) synergistically act to reduce the expression of key angiogenic and metastatic factors (including VEGF, bFGF, MMP-2, and MMP-9) in VBL-treated colon cancer cells, reducing LoVo cell invasion. The VBL/AST combination results in sustained tumor regression	Hirao et al. (1986), Liu et al. (2016), Auyeung et al. (2014), Wang et al. (2023a)
	D: Distributed in the bloodstream, with higher levels in the thymus and spleen			
	M: Metabolized in the liver via hydrolysis, oxidation, and reduction; undergoes glycosylation, demethylation, and hydroxylation in intestinal feces			
	E: Excreted via hepatic bile or renal pathways			
Saikosaponin	A: Absorbed into the bloodstream as original components or as intestinal metabolic products, secondary glycosides, and sapogenins	The structural characteristics of the molecule are closely related to its bioavailability. The hydrophilic nature of Saikosaponin D leads to poor absorption in the human gastrointestinal tract	The combined use of Doxorubicin (Dox) and Saikosaponin D inhibits tumor growth and P-gp expression more effectively than using Dox or Saikosaponin D alone	Li, 2017; Guo et al. (2018), Tang et al. (2020), Zhou et al. (2021)
	D: Exhibits the highest exposure in the liver and prehepatic regions			
	M: Metabolized in the gastrointestinal tract and liver, involving stepwise hydrolysis of glycosides and dehydrogenation, hydroxylation, and carboxylation of sapogenins			
	E: Primarily filtered by the kidneys and excreted in urine			
Diosgenin	A: Absorbed as secondary sapogenins after gastrointestinal transformation	Highly hydrophobic, resulting in extremely low oral bioavailability and poor absorption in the small intestine	Synergistic interaction between Chlorbet and Diosgenin inhibits Chlorbet-induced increase in liver cholesterol synthesis, while maintaining the effectiveness of Diosgenin in reducing cholesterol absorption	Li et al. (2005), Li, 2017; Wang et al. (2018), Wang et al. (2021b), Zhou et al. (2021)
	D: Extensively distributed in the bloodstream and various tissues			
	M: Metabolized by gut microbiota, sugars hydrolyzed stepwise, metabolized to more easily absorbed sapogenins			
	E: Weak contribution from enterohepatic circulation, primarily excreted in feces			
Pulsatilla Saponin	A: A minor portion directly absorbed in its original form	Molecular structure impedes passage through intestinal cell membranes	Pulsatilla Saponin combined with 5-Fluorouracil reduces B-cell lymphoma 2 protein expression and promotes overexpression of miR-24-3p, targeting and downregulating RNF2 expression, inhibiting tumor cells	Zhou, 2022; Li et al. (2020), Ye et al. (2023), Li, 2017
	D: Extensively distributed to tissues, highest concentrations in the kidneys			
	M: Deglycosylated by gut microbiota, resulting in shorter sugar chains			
	E: Mainly metabolized in the liver, then excreted by the kidneys			
Platycodin saponin	A: Hydrolyzed in the gastrointestinal tract to secondary sapogenins	Gastrointestinal metabolism and first-pass effect result in low bioavailability and prolonged absorption of Platycodin D	Combination of Simvastatin, Platycodin G, and Platycodin D enhances LDLR expression and LDL-C uptake in HepG2 cells, showing synergistic effects	Kwon et al. (2017), Li, 2017
	D: Distributed in the blood and various tissues			
	M: Undergoes acetylation, deglycosylation, and desulfation in the gastrointestinal tract			
	E: Predominantly excreted through bile			
Licorice saponin	A: A small portion absorbed in its original form post oral administration	Large molecular weight and low solubility hinder effective cellular membrane passage	Application of Glycyrrhizic acid diethyl ester to the intestines improves absorption of Licorice saponin	Hirao et al. (1986), Tao et al. (2013), Yi et al. (2022)
	D: Distributed in the blood and various tissues			
	M: Undergoes Phase I and Phase II metabolic reactions in the liver, hydrolyzed by bacterial strains in different parts of the intestines			
	E: Excreted through kidneys and bile			
gynostemma saponin	A: A minor portion directly absorbed, most require gastrointestinal transformation	Chemical structure, including types, numbers, and attachment positions of sugar moieties	—	Chen et al. (2015b), Zhao et al. (2022)
	D: Distributed in the blood and various tissues			
	M: Active components metabolized by gut microbiota to produce final products			
	E: Mainly metabolized by the liver, then excreted by the kidneys			

## 3 Pharmacokinetics of saponins

### 3.1 Absorption, distribution, metabolism, and excretion (ADME) of saponins

#### 3.1.1 Absorption

The pharmacokinetic journey of saponins within the human body, particularly their absorption phase, is fraught with challenges. These naturally occurring compounds display inherently low absorption rates through the gastrointestinal (GI) tract, a phenomenon that can be attributed to their unfavorable physicochemical characteristics. Specifically, their substantial molecular weight (exceeding 500 Da), propensity for extensive hydrogen bonding (with a capacity greater than 12), and significant molecular flexibility (with degrees of freedom surpassing 10) collectively contribute to diminished membrane permeability. Moreover, the swift and extensive excretion of saponins via the bile route markedly limits their oral bioavailability, a fact underscored by numerous studies (Yu et al., 2012).

Emerging research highlights the potential of hydrolyzed saponin derivatives, or ‘rare saponins’, to surmount these absorption barriers. These derivatives, generated through various biotransformation mechanisms—ranging from physical methods (heating) and chemical processes (acid hydrolysis) to microbial actions—exhibit structural alterations that favor absorption (He et al., 2019). Notably, the engagement of the gut microbiota in the microbial transformation of saponins, primarily through the cleavage of saponin sugar chains, transforms these compounds into rare saponins with shortened sugar chains. This not only augments their bioavailability but also frequently enhances their biological efficacy relative to that of their natural counterparts (Joaquín et al., 2018).

Intriguingly, studies exploring the intestinal absorption dynamics of saponins, such as the investigation of sea cucumber saponins by Li et al., have illuminated the transport and permeability characteristics of these compounds. Their findings revealed that specific saponins, including holotoxin A₁ and saikosaponin A (SSa), are absorbed via passive diffusion—a process seemingly unaffected by P-glycoprotein (P-gp) efflux mechanisms (Li et al., 2016). Additionally, the work of Xu et al. on the absorption of Panax notoginseng saponins encapsulated within chitosan nanoparticles in rat intestines provides compelling evidence of the enhanced intestinal absorption achievable through nanoencapsulation techniques, as evidenced by high-performance liquid chromatography (HPLC) analyses (Xu et al., 2022). This advancement underscores a significant leap over traditional formulations and raw materials, promising a new horizon for saponin-based therapeutics.

#### 3.1.2 Distribution

The distribution of saponins within the body is a pivotal aspect of their pharmacokinetics. Guo et al. employed high-performance liquid chromatography coupled with tandem mass spectrometry (HPLC-MS/MS) to measure the levels of Saikosaponin B4, Hederagenin, and 23-hydroxybetulinic acid in rat plasma and liver tissues. Their findings revealed that 23-hydroxybetulinic acid, although present in lower quantities, exhibited the highest exposure in both the liver and prehepatic regions (i.e., portal vein plasma and systemic plasma) following hepatic processing. Saikosaponin B4 had the highest initial concentration in the herbal medicine, followed by Hederagenin, which has relatively limited exposure. The hepatic first-pass effect was 23% for 23-hydroxybetulinic acid, 27% for saikosaponin B4, and 3% for Hederagenin, reflecting their varying degrees of hepatic distribution (Guo et al., 2018).

Raw polysaccharide (RPS) is metabolized by enzymes such as α-glucosidase and β-glucosidase in the gut microbiota of rats, resulting in the formation of diosgenin (Li et al., 2005). This metabolite was detected in various tissues, and oral administration of RPS led to prolonged retention of diosgenin in the intestines. Despite the absence of significant enterohepatic circulation in rats, RPS has demonstrated extensive distribution across various body tissues over an extended period (Li et al., 2005). Following oral administration of RPS, the highest concentrations of protopanaxatriol, protopanaxadiol, and their derivatives were observed in the spleen, with levels 50–300 times greater than those in blood saponins. Intriguingly, diosgenin, a hydrolysis product, is predominantly found in the lungs and brain (Wang et al., 2018). The elevated concentration of diosgenin in the brain underscores its potential role as an antinociceptive agent, particularly in chronic cancer pain models. The substantial distribution of saponins in the liver and lungs suggests their therapeutic potential for treating liver and lung diseases. Notably, diosgenin exhibited the slowest metabolic rate in the brain; 144 h after the last oral dose of RPS, its tissue concentration in other areas decreased to 3%, while it maintained approximately 20% in the brain (Wang et al., 2021).

#### 3.1.3 Metabolism

The metabolic pathway of orally administered ginsenosides is intricate and involves multiple stages. Initially, these compounds navigate through the stomach and small intestine, where they remain stable against the effects of gastric acid and local enzymes. Upon reaching the large intestine, ginsenosides are subjected to deglycosylation by the colonic microbiota. This process involves the sequential detachment of oligosaccharides from the aglycone core, beginning with terminal sugars, leading to the generation of key metabolites such as 20S-protopanaxatriol 20-O-β-D-pyran glucose glycoside (M1) and 20S-protopanaxatriol (M4). These metabolites further undergo esterification with fatty acids, forming fatty acid conjugates with enhanced persistence in the body compared to their precursor molecules. It is posited that ginsenosides act as prodrugs, necessitating microbial deglycosylation and subsequent esterification within the body for their activation (Hasegawa, 2004).

In the liver, specifically within hepatocytes, ginsenosides are metabolized via cytochrome P450 enzymes. Using high-performance liquid chromatography coupled with hybrid ion trap and time-of-flight mass spectrometry, researchers have determined that the primary metabolic pathway for PPT ginsenosides in human and rat liver microsomes involves modifications of the C20 aliphatic side chain. Ginsenosides such as Rh1, Rg2, Rf, and PPT predominantly undergo metabolism through this pathway, with CYP3A4 being the principal enzyme responsible for their oxidative transformation. The intrinsic clearance rates of these ginsenosides are ranked as follows: Rf ≤ Rg2 < Rh1 < PPT (Hao et al., 2010).

Furthermore, Guo et al. identified and relatively quantified saponins and their metabolites in rat feces. Their study revealed eleven Panax notoginseng saponin (PNS) metabolites in the normal control group, indicating that hydrolysis and dehydration are the primary biotransformation pathways for PNSs. A comparative analysis between the normal control group and the germ-free (GF) group highlighted the vital role of the gut microbiota in the *in vivo* biotransformation of PNSs. In total, 43 PNS-derived compounds, including 38 flavonoids and five ginsenosides, were detected in rat bile. This study demonstrated that HSYA and ginsenosides predominantly remain in their prototype forms in rat bile, whereas flavonoid glycosides from SF are metabolized into phase II conjugates in bile. Notably, compared with the administration of the individual extracts, the combined administration of PNS-related components did not significantly alter the metabolic profile (Guo et al., 2020).

#### 3.1.4 Excretion

The excretion of ginsenosides, particularly ginsenosides Ra 3) and Rb 1), primarily occurs via passive biliary pathways. Furthermore, the active biliary excretion of ginsenoside Rd is notably slower than that of other ginsenosides. This slow biliary excretion, coupled with inefficient metabolism and sluggish renal clearance, results in the prolonged circulation of these ginsenosides, thereby leading to relatively higher exposure levels in the body. Consequently, the ginsenosides Ra 3), Rb 1), and Rd have been identified as key pharmacokinetic markers that reflect systemic exposure to Panax notoginseng extracts in rats (Liu et al., 2009). In an effort to quantify ginsenoside Rd in biological samples, Cui et al. harnessed an advanced gas chromatography‒mass spectrometry (GC‒MS) technique complemented by rapid solid-phase extraction using a novel C18 microcolumn. This approach facilitated the recovery of more than 80% of the ginsenoside Rd from urine samples, demonstrating an intra-assay variability of less than 5.0%. Subsequent to administering a single dose of a Panax formulation, the study revealed a linear relationship between the intake of ginsenoside Rd and urinary excretion of 20(S)-protopanaxatriol glycoside, with excretion rates ranging from 0.2% to 1.2% in human urine (Cui et al., 1997). Moreover, Zhou et al. revealed that total saponins from Dioscorea nipponica (TDN) could effectively reduce serum uric acid levels in hyperuricemic rats. This reduction was achieved by regulating the abnormal expression of renal organic anion transporters, namely, rOAT1, rOAT3, and rURAT1. In a study involving repeated oral administration of 15 mg/kg diosgenin for 28 days, a noticeable increase in the half-life (T1/2) was observed in rats. This finding indicates a potential accumulation phenomenon and a dose-dependent trend in the metabolism and excretion of diosgenin (Zhou et al., 2015).

### 3.2 Pharmacokinetics and dose‒exposure relationships of saponins

In terms of dose‒exposure relationships, studies have shown correlations between different saponins and dosage. For example, research on the pharmacokinetics of different doses of SSa in Wistar rats revealed a nonproportional increase in systemic exposure to SSa based on AUC values, indicating dose-dependent pharmacokinetics of SSa (Fu et al., 2019) and highlighting the complexity of its pharmacokinetic behavior. In a meticulously designed single-dose study employing a randomized, open-label, three-way crossover design, subjects received ginsenoside Rd intravenously at doses of 10, 45, or 75 mg, with a 2-week washout period between each dosing session. The study revealed that the maximum concentration (C (max)) and AUC of Rd increased linearly and proportionally with increasing dose, indicating predictable pharmacokinetics at these dosages. Notably, the average half-life (t (1/2Z)) ranged from 17.7 to 19.3 h, and the clearance rate (CL/F) between 0.36 and 0.39 L/h remained consistent across doses, underscoring the stable pharmacokinetic profile of ginsenoside Rd regardless of the administered dose. Furthermore, a multiple-dose regimen involving the daily administration of 10 mg of Rd for six consecutive days resulted in a modest accumulation of the drug. The observed mean peak concentration at steady state (C (max)), total AUC from time zero to infinity (AUC (0-∞)), and steady-state AUC (AUC (ss)) were 4.0 mg/L, 51.7 mg x h/L, and 26.4 mg x h/L, respectively. The half-life (t (1/2Z)) under this regimen was reported to be 20.5 h, aligning closely with the findings from a single-dose study. Ginsenoside Rd was well tolerated across the studied doses, with no evident pattern of dose-related adverse events, demonstrating its safety profile (Zeng et al., 2010). Moreover, Sun et al. explored the pharmacokinetics of a 15 mg/kg dose of Triterpenoid Saponin D (TED) administered orally to rats over a 28-day period. The study noted an elongated half-life (T1/2) for the 15 mg/kg TED dose, indicating both an accumulation phenomenon and a dose-dependent trend in pharmacokinetics. This finding adds to the growing body of evidence suggesting the importance of considering dose-dependent effects in the pharmacokinetic modeling of saponins (Sun et al., 2022).

### 3.3 Factors influencing the pharmacokinetics of saponins

#### 3.3.1 Formulation factors

The pharmacokinetic behavior of saponins, including their absorption, distribution, metabolism, and excretion, is significantly impacted by their administration route. Due to the inherent low solubility of saponins and their role in activating P-glycoprotein (P-gp), these compounds exhibit poor intestinal absorption and notably low bioavailability (Li et al., 2021). Modifying the dosage and solvent has been shown to substantially enhance intestinal absorption (Pan et al., 2018). Moreover, the pharmacokinetics of saponins can vary greatly depending on the method of administration. Intravenous administration leads to rapid elimination from the body, whereas oral administration may involve enterohepatic circulation, affecting the duration that saponins remain in the system. This dichotomy suggests that while injectable forms of saponins can be developed into slow-release and controlled-release preparations for clinical use, oral preparations may be less favorable due to their pharmacokinetic disadvantages.

To counteract these challenges, it is crucial to optimize the formulation process by considering the characteristics of biological utilization. This includes altering the route of administration and formula composition, as well as incorporating modern pharmaceutical technologies to create innovative drug delivery systems. Examples of such systems include liposomes, nanoparticles, and solid dispersions, which aim to improve the pharmacokinetic profiles of saponins (Kim, H. et al., 2018). A notable study by Liu et al. (2013) focused on the development of a solid dispersion (SD) formulation for raw polysaccharide (RPS) saponins. This study evaluated seven different carriers for SD, identifying poloxamer 407 (P 407) as the most suitable due to its ability to reduce the particle size of saponins in aqueous solutions, increase their solubility by approximately 3.5-fold, and significantly enhance the absorption and transport of saponins in rat intestinal sacs—from 48 μg to 104 μg. This advancement underscores the potential of solid dispersions for improving the bioavailability and overall pharmacokinetic profile of saponins (Liu et al., 2013).

#### 3.3.2 Saponin intrinsic structural factors

The pharmacokinetic profiles of ginsenosides are profoundly influenced by their chemical structures, particularly their stereochemistry. Bae et al. revealed that the 2(S)-epimers of ginsenosides Rg20 and Rh3 exhibit greater permeability and oral absorption than their 20(R)-epimers, as indicated by higher peak plasma concentrations (C.max) and greater area under the curve (AUC) values. This evidence highlights the critical impact of stereochemistry on the absorption and overall pharmacokinetics of saponins (Bae et al., 2013). Moreover, the structural details of saponins significantly dictate their pharmacokinetic behaviors. For example, despite their structural similarities, ginsenosides Rb1 and Rb2 show distinct oral absorption rates. The oral absorption of ginsenoside Rb1, which contains a hexose and hydroxyl group, is superior to that of ginsenoside Rb2, which contains a pentose group. The oral bioavailability of ginsenoside Rb1 in rats is estimated to be approximately 0.78%, in stark contrast to the 0.08% observed for ginsenoside Rb2 (Zhao et al., 2012). This variance underscores the influence of molecular structure on the bioavailability of saponins.

The antioxidant activity of ginsenosides is also believed to be influenced by the type of sugar molecule they contain. Ginsenosides Rb1 and Rc, which feature disaccharide glycosidic bonds, exhibit more substantial antioxidant activity than ginsenoside Rb20, which has a monosaccharide glycosidic bond. This suggests that the glucose-arabinose bond at the C-28 position may play a crucial role in enhancing the antioxidant properties of ginsenosides. Furthermore, the position of the sugar moiety significantly affects the pharmacological effects of ginsenosides. Those with sugar moieties at the C-3 or C-20 position are found to have greater pharmacological activity than those with sugar moieties at the C-6 position (Li et al., 2009).

#### 3.3.3 Physiological factors

The implementation of precision drug therapy necessitates an understanding of individual variability in pharmacokinetics (PK), particularly because TCM often displays pronounced species differences within the body (Bechtold and Clarke, 2021). For instance, the ginsenoside protopanaxadiol (20(S)-protopanaxadiol), which is derived from the stem and leaves of *Panax notoginseng*, has been shown to undergo distinct metabolic processes in human and rat liver microsomes, illustrating the complexity of species-specific metabolic pathways (Martinez and Staba, 1984). In an investigation into the plasma protein binding characteristics of 20(R)-Rh2, equilibrium dialysis was used to assess the binding fraction at four different concentrations (50, 100, 200, and 400 ng/mL) in both rat and human plasma. The study revealed substantial species differences, with a binding fraction of approximately 70% in rats compared to approximately 27% in humans across all tested concentrations. This disparity underscores the significant variability in plasma protein binding between species (Gu et al., 2006). By further exploring species-specific pharmacokinetic differences, Zhou CQ evaluated alterations in blood concentration over time and the bioavailability of Pulsatilla saponin B4 following rectal and injection administration in Japanese rabbits. This study revealed that rectal administration resulted in a peak concentration (C_max_) of 3.03 ng/mL, which was significantly lower than the 83.8 ng/mL observed with injection. Moreover, the half-life (T1/2) was 2.76 h for rectal administration and 3.372 h for injection. A comparative study in SD rats demonstrated a longer half-life for rectally administered drugs than for those administered via injection, with a bioavailability of 1.46% for the rectally administered group (Maroyi, 2018). These findings highlight the distinct pharmacokinetic profiles of saponin B4 from Pulsatilla chinensis in Japanese rabbits *versus* SD rats, emphasizing the impact of biological species variations on drug metabolism and bioavailability.

#### 3.3.4 Environmental factors

The efficacy and consistency of TCM are profoundly influenced by a myriad of environmental factors, rendering the prescription process fraught with variability. On the one hand, the inherent quality of TCM at any given time is shaped by an array of elements, including but not limited to climatic conditions, topographical variations, soil composition, and biological influences (Liu et al., 2020). These factors can affect the growth, potency, and composition of medicinal plants, leading to variability in the raw materials used for TCM preparations.

On the other hand, the various stages of TCM production and preparation, such as planting, harvesting, and storage, are also subject to the impact of environmental factors. These stages are critical in determining the final quality and therapeutic potential of TCM products (Fan et al., 2022). Variations in environmental conditions during these phases can induce significant differences in the pharmacokinetic profiles of TCM compounds. As such, these environmental variances introduce substantial challenges in achieving uniformity in TCM quality and complicate efforts to summarize and predict pharmacokinetic regularities within TCM research.

The influence of environmental factors extends beyond mere production challenges; it also encompasses the broader issue of maintaining and ensuring the therapeutic integrity of TCM across different batches and production cycles. Given the sensitivity of TCM components to their growth and processing conditions, the pharmacokinetic properties of these medicines can fluctuate, potentially affecting their efficacy and safety profiles. This indicated the necessity for rigorous quality control measures and sophisticated analytical methods to assess and mitigate the impact of environmental variability on TCM pharmacokinetics.

### 3.4 Interaction of saponins with other drugs

#### 3.4.1 Enzyme inhibition and induction

Jiang et al. found that curcuminoid VI improves depression-like behavior in CMS mice based on the SPT, TST, and FST, which is associated with the transition of glial cells in the hippocampus from a proinflammatory phenotype (iNOS-Iba1) to a neuroprotective phenotype (Arg-1-Iba1). Chronic mild stress (CMS) reduces the expression levels of PPAR-γ and phosphorylated PPAR-γ in the hippocampus. Curcuminoid VI partially reversed this effect. GW9662 treatment blocked the nuclear translocation of PPAR-γ in microglia treated with Curcuminoid VI and suppressed the induction of Arg-1 in microglia. Blocking PPAR-γ signaling also eliminated the ability of Curcuminoid VI to inhibit proinflammatory cytokines while increasing anti-inflammatory cytokines in the hippocampus of CMS mice. Curcuminoid VI induces a neuroprotective microglial phenotype in the hippocampus through the PPAR-γ pathway, thereby improving CMS-induced depression-like behavior (Jiang et al., 2022). Tanshinone IIA exhibits cytotoxicity to tumor cells by inhibiting mTOR and inducing ER stress. Inhibition of mTORC1 is characterized by a significant reduction in the phosphorylation of mTORC1 targets. Furthermore, it induces endoplasmic reticulum stress, leading to the phosphorylation of eIF2α and activation of caspase-4. These proapoptotic pathways are selectively activated by tanshinone IIA in tumor cells but not in normal cells (King et al., 2009). The aldo-keto reductase inhibitory activity of Balanites fruit is attributed to the presence of steroidal saponins. HPLC chromatograms of the crude butanol fractions and their four subfractions revealed that the biologically most active fraction, D, contained the greatest amount of steroidal saponins (21%), compared to 75% in the original butanol fraction. The isolated furostanol saponins demonstrated high activity in vitro assays. The more polar parts of Balanites fruits containing saponins (BuFr and RwFr) showed greater aldo-keto reductase inhibitory activity (Abdel Motaal et al., 2015). Panax notoginseng saponin (PNS) induces CYP1A2, resulting in decreased caffeine C (max) (36.3%, *p* < 0.01) and AUC(0-∞) (22.77%, *p* < 0.05) and increased CL/F (27.03%, *p* < 0.05). Protein blot analysis revealed that the CYP1A2 protein was upregulated by PNS. No significant changes in the activities of CYP2C9, 2D6, or 3A4 were observed. These results suggest that PNS can induce CYP1A2, potentially impacting the disposition of drugs, which is primarily dependent on the CYP1A2 pathway (Liu et al., 2012). Wu et al. isolated eight oleanane-type saponins, including five new saponins, from the roots of Helleborus thibetanus. The isolates were evaluated for their α-glucosidase inhibitory activity. All pentacyclic triterpenoid saponins exhibited significant inhibitory activity, with IC50 values ranging from 18.7 to 154.3 μM, which was markedly greater than that of the positive control acarbose (IC50 = 190.5 μM). Compounds 1 and 2 displayed excellent inhibition of α-glucosidase, potentially contributing to the further development of α-glucosidase inhibitors (Wu et al., 2017).

#### 3.4.2 Regulation of drug transport proteins

Molecular docking and immunoprecipitation studies indicated that paeonol triterpenoid saponins (PTSs) disrupt the Keap2/Nrf1 interaction by directly blocking the binding site of Nrf2 to the Keap1 protein. *In vivo*, PTS reduces the myocardial infarct area in ischemia/reperfusion (I/R) rats and alleviates pathological damage. PTS decreases the activity of myocardial injury markers. PTS regulates Nrf2 nuclear translocation, and ML385 inhibits the therapeutic effect of PTS both *in vitro* and *in vivo*. These results suggest that PTS has therapeutic potential for myocardial ischemia/reperfusion injury (MIRI) by targeting Keap1/Nrf2 activity (Yao et al., 2022). Xu et al. reported that astragaloside IV (AS-IV) significantly inhibits IL-13- and IL-4-induced M2 polarization of macrophages, leading to reduced expression of CD206 and M2-associated genes. AS-IV also inhibited the invasion, migration, and angiogenesis of A549 and H1299 cells induced by M2-CM. *In vivo* experiments showed that AS-IV greatly suppressed tumor growth and reduced the number of Lewis lung carcinoma metastases. The percentage of M2 macrophages in tumor tissue decreases after AS-IV treatment (Xu et al., 2018). Furthermore, AS-IV inhibited AMPKα activation in M2 macrophages, and silencing AMPKα partially abolished the inhibitory effect of AS-IV. These findings suggest that AS-IV could be used as a treatment for myocardial ischemia/reperfusion injury patients (Xia et al., 2015).

#### 3.4.3 Clinically relevant drug interaction

The potential for clinically relevant drug interactions involving saponins has garnered significant research interest. Understanding these interactions is crucial for optimizing therapeutic outcomes and minimizing adverse effects.

Tian et al. conducted a study to examine the interaction between notoginsenoside and aspirin *in vivo*. Their research indicated that coadministration of these drugs in rats led to increased concentrations of five notoginsenoside components, suggesting enhanced absorption when both drugs were used concurrently. This effect was attributed to aspirin and salicylic acid disrupting tight junction proteins, thereby opening intercellular gaps and facilitating the absorption of notoginsenoside. Furthermore, over a 1-year follow-up period, notoginsenoside was found to significantly augment the platelet inhibition rate of clopidogrel in patients undergoing percutaneous coronary intervention (PCI), resulting in fewer major adverse events (MAEs). A clinical trial involving 1200 PCI patients revealed that Panax notoginseng saponins (PNSs) notably improved the platelet inhibitory efficacy of clopidogrel and aspirin, reducing the incidence of major adverse cardiac events (MACE) from 7.8% to 3.3% (Tian et al., 2018). In another study, Liang et al. discovered that Schisandrin Lignans Enhancer (SLE) could increase the exposure of ginsenoside Rb2 by inhibiting the activity and expression of P-glycoprotein (P-gp), thereby decreasing its efflux rate in Caco-2 and L-MDR1 cells. This finding highlights the potential of SLE to enhance the bioavailability of drugs that are P-gp substrates (Liang et al., 2014). Furthermore, Jeon et al. explored the effects of Rb2 on organic anion-transporting polypeptide (OATP/OATP) transport proteins, which are crucial for the hepatic uptake of various drugs. Despite Rb2 demonstrating high inhibitory activity against OATP1B3 and OATP1B1 *in vitro*, *in vivo* studies in rats have shown limited pharmacokinetic changes in valsartan (an OATP substrate) following repeated oral administration of ginseng products. This discrepancy between *in vitro* and *in vivo* findings suggests that the actual risk of herb-drug interactions between Rb2 and OATP substrates may be low, possibly due to the limited blood concentration and hepatic distribution of Rb2, which does not reach the inhibitory concentration (IC) required to affect OATP activity (Jeon et al., 2020).

### 3.5 Pharmacokinetic application of saponins

#### 3.5.1 Pharmacokinetic applications of saponins in drug development

Hederagenin (HG), a triterpenoid saponin, has demonstrated promising antitumor activity, positioning it as a potential chemotherapeutic agent. Zeng et al. reported that structural modifications at various positions (C-3, C-12, C-13, C-23, and C-28) on the HG scaffold resulted in compounds with enhanced potency compared to HG itself. Additionally, strategies to mitigate or eliminate hemolysis through structural modification or formulation design are key to facilitating the transition of HGs from preclinical stages to clinical research, potentially accelerating their development as cancer therapeutics (Zeng et al., 2018). Ginsenosides, which have properties such as low solubility, poor stability, short half-life, easy elimination, and degradation *in vivo*, face limitations in clinical application. The development of delivery systems for ginsenosides used as dual-function drugs or carriers is essential. To develop precise cancer treatment strategies, various nanodelivery systems and preparation techniques based on ginsenosides, including polymer nanoparticles (NPs), liposomes, micelles, microemulsions, protein NPs, metal and inorganic NPs, and biomimetic NPs, have been developed. Additionally, the unification of ginsenoside-based drugs and carriers is crucial for enhancing drug bioavailability and targeting capability (Wang et al., 2021). Research has shown that the ginsenoside Rg1 can improve spatial learning and memory in AD animal models. The administration of ginsenoside Rg1 obtained through Morris water maze (MVM) testing shortened the path length of platform search in model rats but had no effect on the path length of sham-operated rats (Shi et al., 2012), which indicates that ginsenoside Rg1 only alleviates memory impairment in AD animals and does not cause changes in normal animal indices in the same test. Rg1 has the potential to be developed as a therapeutic drug for Alzheimer’s disease (Wu et al., 2022). Zhu et al. reported that the liposome system of ginsenoside Rg3 (Rg3-LPs) not only significantly improved the uptake and infiltration of glioma spheres by cells *in vitro* but also significantly enhanced the targeting and *in vivo* diffusion ability of active gliomas. Rg3 is a good substitute for cholesterol in drug delivery liposomes and has synergistic effects with loaded anticancer drugs. Rg3-PTX-LPs can serve as multifunctional potential drugs for the treatment of glioma (Zhu et al., 2021).

#### 3.5.2 Pharmacokinetic applications of saponins in clinical treatment

Chen et al. conducted a pivotal study on the efficacy of Rh2, a dammarane saponin derived from ginseng, in treating dextran sulfate sodium (DSS)-induced colitis, a model for ulcerative colitis (UC). Rh2 significantly mitigated DSS-induced symptoms, including weight loss, intestinal damage, colon shortening, and disease activity index (DAI) changes. Additionally, Rh2 administration led to a notable reduction in the levels of proinflammatory cytokines such as TNF-α, IL-6, and IL-1β, highlighting its anti-inflammatory potential. This study also revealed that Rh2 inhibited the activation of STAT3/miR-214, a pathway implicated in the inflammatory response associated with UC. These findings underscore the therapeutic value of Rh2 in UC treatment through the modulation of the STAT3/miR-214 pathway (Chen et al., 2021). In another study, Choi et al. evaluated the pharmacokinetics of repeated administration of high-dose red ginseng over 15 days, demonstrating good tolerance and stable plasma concentrations of key ginsenosides, including Rb2, Rb15, and Rc. Notably, after 15 days, the area under the concentration-time curve (AUC) values for these ginsenosides were 4.5–6.7 times greater than those for a single dose. The study also revealed significant interindividual variability in the plasma concentrations of Rd and compound K, yet a strong correlation between their AUC values suggested a consistent metabolic pathway from Rb1, Rb2, and Rc to Rd and subsequently to compound K. These findings highlight the potential for optimizing biotransformation strategies to increase the plasma concentrations of these metabolites, thereby improving the therapeutic efficacy of red ginseng (Choi et al., 2020).

In summary, the pharmacokinetics of saponins are indispensable for the development of new saponin drugs. In recent years, there have been an increasing number of reports on the pharmacokinetics of TCM saponins, and research methods have also begun to take shape. The emergence of new theories and ideas has also provided breakthrough progress for more accurate and scientific disclosure of the pharmacokinetic characteristics of TCM saponins. In summary, the current research on the pharmacokinetics of TCM saponins has focused mainly on the inherent active ingredients of TCM, and *in vivo* research on saponin secondary metabolites is still limited. Therefore, further studies of the pharmacokinetics of combinations of metabolites and active ingredients can provide new ideas for revealing the material basis and mechanisms of TCM efficacy.

## 4 Challenges and future directions

### 4.1 Current challenges in research

#### 4.1.1 Limitations of pharmacokinetic research methods

TCM, characterized by its multicomponent nature (Fu et al., 2018). Presents a critical challenge in selecting which specific chemical components or classes should serve as representative indicators for the entire formulation (Wu et al., 2018; Sun et al., 2020). The complexity of Chinese herbal components makes it difficult to determine suitable indicators for pharmacokinetic studies (Yang et al., 2020). In cases where certain active components are present at low concentrations, it is often necessary to use concentration methods after sample separation and extraction to enrich the targeted components for analysis (Jing and Gu, 2023). The diversity of TCM sources and varieties leads to a wide range of water content characteristics, which may result in the generation of structurally similar compounds during the extraction and separation processes, potentially affecting sample analysis. The specificity of pharmacokinetic studies on TCM is evident in the fact that the same herbal medicine may be used in different prescription environments. Moreover, herbal medicines of the same origin often undergo different harvesting seasons and various processing techniques (Ma et al., 2016). Consequently, the pharmacokinetic parameters of the same herbal component can be influenced. This is a significant difference from Western medicines, as it entails variations in the absorption rate, extent of absorption, and elimination rate of a given component. Therefore, obtaining reproducible results for the same TCM is relatively more challenging than obtaining reproducible results for Western medicines.

#### 4.1.2 Data interpretation and model establishment challenges

The development of pharmacokinetic modeling for TCM saponins must account for the unique challenges posed by the complexity of these compounds. This includes the construction of physiologically based pharmacokinetic (PBPK) models, which can capture both intrinsic factors (such as organ function variability, age-related changes, and genetic polymorphisms) and extrinsic factors (including drug-drug interactions, environmental conditions, and lifestyle factors). While PBPK models offer comprehensive insights, they are part of a suite of tools necessary for a holistic understanding of TCM saponin pharmacokinetics. Random population models, which utilize default physiological parameters from software such as GastroPlus™, necessitate meticulous calibration to ensure the representation of diverse physiological conditions. This calibration often involves adjusting the coefficient of variation (CV%) to account for the broader variability in human populations. The modeling of special populations, such as those with specific genetic backgrounds, organ impairments, or comorbid conditions, is inherently more challenging. Careful integration of specialized physiological and pharmacokinetic parameters, which may not be readily available from the literature, is needed, especially for novel or less studied saponin compounds (Wang et al., 2023). A significant hurdle in the development and validation of these models is parameterization—the collection and accurate representation of necessary data. While sourcing parameters from published studies, there is an inherent degree of uncertainty in the prediction of outcomes, given the variability in data quality and applicability.

#### 4.1.3 Variability and predictability in clinical applications

Extensive research and development have been conducted on saponins in recent years. However, due to their physicochemical properties, as well as limitations in pharmacokinetics and safety, coupled with limited studies on their pharmacological activity and mechanisms of action, the clinical application of saponins still faces considerable challenges. Saponins exhibit relatively low bioavailability, poor water solubility, and short half-lives after oral absorption. As a result, frequent dosing of saponin compounds leads to fluctuations in plasma drug concentrations and increases the risk of unfavorable clinical outcomes, including side effects and adverse reactions, restricting their clinical application. These drugs may cause nonlinear hepatic first-pass effects. There may be significant clinical differences between the models. A model is needed to quantitatively predict the impact of changes in hepatic blood flow, hepatic intrinsic clearance, or drug-protein binding on blood drug concentrations. The models may exhibit clinically significant differences in predicting these changes. The most significant differences lie in predicting the impact of changes in hepatic blood flow for orally administered drugs on blood concentrations, as well as the effect of protein binding on the concentration of unbound blood drugs (Morgan and Smallwood, 1990). Zhou et al. (2022) comprehensively assessed the predictive performance of models through various aspects, including prediction error (PE), mean prediction error (MPE), normalized prediction distribution error (NPDE), and Q‒Q plots. In the external validation cohort, patients from the same center exhibited more similar population characteristics, such as disease spectrum distribution. Therefore, the predictive ability of the model was the highest. When stratified by different indicators, the prediction error of each model varied. After stratification by infection site, most models demonstrated good applicability in patients with central nervous system infections.

### 4.2 Future directions

#### 4.2.1 Advanced pharmacokinetic investigations of novel saponin derivatives

Innovative approaches in pharmacokinetic studies of saponins are paving the way for enhancing their therapeutic efficacy and addressing existing limitations in their clinical application. The pioneering work by Jin et al. introduced a groundbreaking colonic delivery system specifically designed for ginsenosides, achieving targeted release directly into the colon and maintaining constant release rates, thereby significantly boosting the effectiveness of these compounds. The system showed almost perfect zero-order kinetics in releasing ginsenosides within an 8-h timeframe at an alkaline pH of 12.7, according to *in vitro* dissolution assessments. The success of this optimal formulation was shown to be largely independent of device configuration or agitation velocity, relying instead on the osmotic gradient across the membrane. *In vivo* evaluations in beagle dogs revealed a marked enhancement in both the bioavailability and retention time of the ginsenosides compared to standard formulations, underscoring the potential of this colon specific, osmotically driven system to bypass gastrointestinal irritation, reduce administration frequency, stabilize plasma concentration levels, and improve oral uptake (Jin et al., 2018). In a subsequent study, Jin and collaborators developed berberine vesicle particles augmented with natural ginsenosides to increase dissolution rates and potentiate the antiplatelet clustering effects of berberine. These modified nanoparticles were synthesized using a method combining homogenization and lyophilization, with ginsenosides acting as surfactants to prevent particle aggregation by electrostatic repulsion and further stabilized by trehalose during freeze-drying. Analytical techniques confirmed the preservation of the postmodification crystalline structure postmodification, enhancing its solubility upon reconstitution. Pharmacokinetic analysis revealed a more than fourfold increase in bioavailability for the modified nanoparticles compared to unmodified berberine vesicles, with significant improvements in inhibiting platelet aggregation attributed to the increased bioavailability of berberine and synergistic effects with ginsenosides (Jin et al., 2019).

#### 4.2.2 Optimization of drug combinations and individualized treatment strategies

Optimizing drug combinations and developing individualized treatment strategies represent pivotal aspects of modern pharmacotherapy, aiming to enhance therapeutic efficacy while minimizing adverse effects. Wang et al. (Wang et al., 2021) studied the combined action of ginsenosides with aspirin and explored the potential mechanisms of arachidonic acid (AA) metabolism through lipidomic analysis. In a randomized, assessor-blind trial, 42 patients with stable coronary heart disease (SCHD) and chronic gastritis were randomly assigned to receive ASA (n = 21) or PNS + ASA (n = 21) for a duration of 2 months. Compared to ASA alone, PNS + ASA further inhibited CD62p expression, GPIIb-IIIa activation, and platelet aggregation, leading to an increased platelet inhibition rate. PNS + ASA suppressed the activity of platelet cyclooxygenase (COX)-1, reducing the production of TXB2, PGD2, PGE2, and 11-HETE, which are downstream oxylipins of the AA/COX-1 pathway in platelets. Compared with patients in the ASA group, patients in the PNS + ASA group experienced alleviation of digestive symptoms, possibly related to increased secretion of gastrin and histamine. This study suggested that the combination of PNS and ASA enhances the antiplatelet effect of ASA through the AA/COX-1/TXB2 pathway and alleviates ASA-related gastric injury through the AA/PG pathway in the gastric mucosa. When warfarin and PNSs were coadministered, the prothrombin time (PT) and international normalized ratio (INR) increased compared to those of patients treated with warfarin alone. After 72 h of administration, PT increased by 110%, 122%, and 126% in the low-dose PNS, medium-dose PNS, and high-dose PNS groups, respectively, compared to that in the warfarin alone group (all *p* < 0.05). PNS increased the blood concentration and exposure time of warfarin and enhanced its anticoagulant effect by inhibiting the expression of the hepatic enzyme CYP3A4 (Qian et al., 2022).

Clinical studies are crucial for obtaining a comprehensive understanding of a drug’s potential mechanisms in the human body. To be truly effective, clinical designs should ensure that participants represent the entire population in a fair manner, including various characteristics such as age, gender, race, disease severity, and other special population classifications. Incorporating the variability in physiological parameters among different special populations into PBPK models can encompass the entire population. Personalized medicine remains a very current and pressing challenge, despite decades of strong interest from researchers and healthcare providers. With advancements in knowledge in this field, the need for physiologically tailored therapies has become increasingly apparent. There are significant physiological and pharmacokinetic differences among patients due to gender, age, race, disease status, and pregnancy (Hartmanshenn et al., 2016).

## 5 Conclusion

Recent advancements in the study of saponins have heralded a new era in pharmaceutical science, characterized by significant strides in drug delivery technologies and therapeutic applications. Innovations such as specialized colonic capsules for targeted release and modified vesicle particles for enhanced bioavailability represent pivotal milestones in the field. The synergistic combination of ginsenosides with aspirin, for instance, illustrates the therapeutic potential of saponins in augmenting the efficacy of conventional drugs. These breakthroughs represent a paradigm shift toward optimizing the bioavailability and effectiveness of saponins, highlighting their integral role in the future of drug development and personalized medicine.

Despite these promising developments, the path from research to practical clinical application requires rigorous clinical validation and further research. This review underscores the critical need for continued exploration into the pharmacokinetics of saponins, leveraging the rich heritage of TCM and modern scientific principles. The study of saponins in TCM, guided by a holistic understanding of their *in vivo* processes and interactions, benefits from the integration of mathematics, biology, chemistry, and advanced analytical technologies. This multidisciplinary approach not only facilitates the development of new clinical drugs but also supports rational clinical medication, offering theoretical and data-driven insights into the utilization of saponin compounds.

TCM, with its profound cultural heritage and repository of natural resources, continues to offer a wealth of medicinal compounds awaiting discovery and application. The ongoing improvement and modernization of TCM research, powered by the identification of effective ingredients and the development of new pharmacokinetic models, are poised to unlock the full potential of these ancient remedies. As we advance, the modernization of TCM and its integration into contemporary pharmacotherapy have become increasingly attainable, promising for enriching the global pharmaceutical landscape with innovative and effective treatment options. The future of saponin research and application in drug development and personalized medicine is bright, with the potential to significantly impact healthcare and patient outcomes. The journey toward fully harnessing the therapeutic potential of saponins is an exciting Frontier in pharmacology, offering hope for new solutions to complex medical challenges.
